#  ZNF185 is a p53 target gene following DNA damage

**DOI:** 10.18632/aging.101639

**Published:** 2018-11-16

**Authors:** Artem Smirnov, Angela Cappello, Anna Maria Lena, Lucia Anemona, Alessandro Mauriello, Nicola Di Daniele, Margherita Annicchiarico-Petruzzelli, Gerry Melino, Eleonora Candi

**Affiliations:** 1 Department of Experimental Medicine, TOR, University of Rome “Tor Vergata”, 00133 Rome, Italy; 2 Department of Systems Medicine, University of Rome “Tor Vergata”, 00133 Rome, Italy; 3 Istituto Dermopatico dell’Immacolata-IRCCS, 00163 Rome, Italy; 4 MRC-Toxicology Unit, University of Cambridge, Cambridge, UK

**Keywords:** p53, ZNF185, DNA damage, cytoskeleton, skin, epithelial cancer

## Abstract

The transcription factor p53 is a key player in the tumour suppressive DNA damage response and a growing number of target genes involved in these pathways has been identified. p53 has been shown to be implicated in controlling cell motility and its mutant form enhances metastasis by loss of cell directionality, but the p53 role in this context has not yet being investigated. Here, we report that ZNF185, an actin cytoskeleton-associated protein from LIM-family of Zn-finger proteins, is induced following DNA-damage. ChIP-seq analysis, chromatin crosslinking immune-precipitation experiments and luciferase assays demonstrate that *ZNF185* is a *bona fide* p53 target gene. Upon genotoxic stress, caused by DNA-damaging drug etoposide and UVB irradiation, ZNF185 expression is up-regulated and in etoposide-treated cells, ZNF185 depletion does not affect cell proliferation and apoptosis, but interferes with actin cytoskeleton remodelling and cell polarization. Bioinformatic analysis of different types of epithelial cancers from both TCGA and GTEx databases showed a significant decrease in *ZNF185* mRNA level compared to normal tissues. These findings are confirmed by tissue micro-array IHC staining. Our data highlight the involvement of ZNF185 and cytoskeleton changes in p53-mediated cellular response to genotoxic stress and indicate ZNF185 as potential biomarker for epithelial cancer diagnosis.

## Introduction

To counteract DNA damage, specific mechanisms have been evolved, these are activated by specific signal transduction pathways, such as the phosphatidylinositol 3-kinase-like protein kinases (PIKKs) family, ATM, ATR and DNA-PK, and the members of the poly(ADP)ribose polymerase (PARP) family [1–7]. Among the effectors, a key role is played by the tumour suppressor protein p53 [8–15]. Indeed, DNA damage leads to p53 stabilisation by inhibition of interaction with its ubiquitin ligase, MDM2 [9], and as consequence, p53 transcriptionally induces cell cycle arrest, apoptosis or senescence. Among p53 targets in response to DNA damage, there are the CDK inhibitor p21, the pro-apoptotic proteins BAX and PUMA [16]. Moreover, p53 directly activates repair pathways such as nucleotide excision repair (NER) through regulation of the NER factors XPC and DDB2 and induces dNTP synthesis [17].

In addition to its roles in cell death, p53 has also been implicated in cytoskeleton assembly, cell motility and mechanosignaling, as negative regulator of cancer cell mobility, invasion and metastasis [18–20]. Integrin expression and signalling pathways, which play a key role in tumour cell invasion and metastasis, have been reported to be regulated indirectly by p53 [18]. For instance, Nutlin-3a, an MDM2 antagonist that acts as p53 activator, decreases the expression of integrin *alpha*5 in colorectal cancer and glioma cells [21,22]; also the expression of integrin *beta*3 decreases upon DNA-damage in wild-type p53 expressing cells [23]. p53 also regulates focal adhesion and Rho signalling pathways by regulating Rho GTPase activity [24] and effector protein genes of RhoA/RhoC and Cdc42 pathways [25–28]. In addition, F-actin formation is negatively or positively regulated by p53 in response to DNA damage depending on the anti-tumour drug used and cell type. For instance, while doxorubicin increases the expression of RhoC and LIM kinase 2 in a p53-dependent manner promoting actin stress fibers formation [29], etoposide and camptothecin attenuate this process through p53-dependent expression of RhoE [30]. It has been also reported that upon etoposide-mediated DNA damage, p53 alters actin cytoskeleton by transcriptionally induction of the expression of the cytoskeleton adaptor protein ankyrin-1 [31]. The relevance of cytoskeleton remodelling and cell mobility in tumours is evidenced by the fact that mutant p53 promotes tumour cell invasion and results in loss of directionality during migration [32]. Cytoskeleton remodelling and cell migration in cancer is a complex process and is controlled by many proteins and pathways, the specific role of p53 in these mechanisms is not yet completely understood.

Here, we describe a novel p53 target gene, *ZNF185*, which codifies for a Zn-finger protein belonging to LIM-family, activated upon genotoxic stress caused by DNA-damaging drug etoposide. ZNF185 itself is not necessary for p53-dependent cell cycle arrest and apoptosis, yet its silencing affects actin cytoskeleton changes and cell polarity upon etoposide treatment. At mRNA and protein level, ZNF185 is strongly reduced in different types of epithelial tumours, including skin and head and neck squamous cell carcinomas, suggesting that depletion of ZNF185 in cancer cells facilitate cancer cell migration and spreading.

## RESULTS

### ZNF185 is a p53 target gene

**Figure 1:**
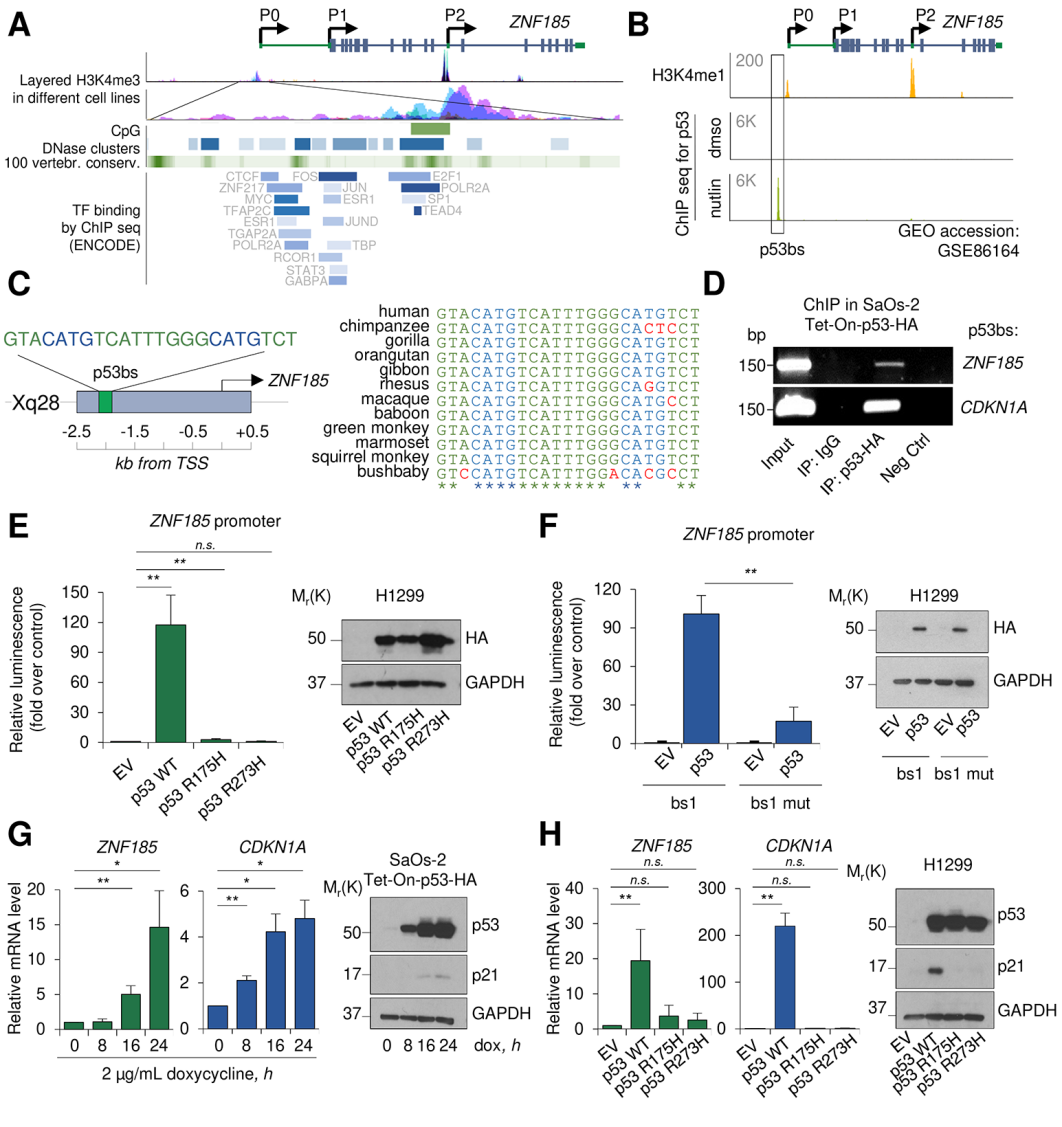
**ZNF185 is a transcription target of p53.** (**A**) UCSC genome browser analysis showing layered H3K4me3 mark in different cell lines, CpG islands, DNase clusters, conservation in vertebrates, and TF binding within *ZNF185* promoter. (**B**) Genomic locus of *ZNF185* showing the promoter region with H3K4me1 and p53 ChIP-seq signals after MCF7 treatment with either DMSO or nutlin. (**C**) Identified p53 binding site (p53 bs) within *ZNF185* promoter region and conservation analysis among primates. (**D**) Amplification of specific DNA fragments after ChIP performed in SaOs-2 Tet-On-p53-HA cells using HA antibody. (**E**) Luciferase activity assay in H1299 after transfection of pGL3-*ZNF185* promoter and either empty vector, p53 WT, p53-R175H, or p53-R273H expression vectors. ** *P*<0.01, *n*=4. Western blot analysis of cell lysates confirms p53 overexpression. (**F**) Luciferase activity assay in H1299 after transfection of pGL3-*ZNF185* promoter with either WT of mutated p53 bs and either empty vector or p53 WT expression vectors. ** *P*<0.01, *n*=3. Western blot analysis of cell lysates to confirm p53 overexpression. (**G**) RT-qPCR analysis of *ZNF185* and *CDKN1A* mRNA levels in SaOs-2 Tet-On-p53-HA after induction of p53 expression with 2 µg/mL doxycycline. * *P*<0.05, ** *P*<0.01, *n*=3. Western blot shows p53 and p21 levels. (**H**) RT-qPCR analysis of *ZNF185* and *CDKN1A* mRNA levels in H1299 after transfection with empty vector, p53 WT, p53 R175H, or p53 R273H expression vectors. ** *P*<0.01, *n*=3. Western blot shows p53 and p21 levels.

We have previously shown that the p53 family member p63, using a novel promoter region and a specific enhancer, directly regulated ZNF185 expression in keratinocytes [33]. To investigate whether also p53 could regulate ZNF185 expression and expand the p53 target genes involved in cytoskeleton regulation and cell polarity, we further analysed *ZNF185* promoter region using UCSC genome browser (Fig 1A). We observed several regions showing high accessibility and conservation between the species, and an enrichment in different transcription factors (TF) binding. Analysis of the publicity available ChIP-seq data for p53 performed in MCF7 cells after p53 stabilization by nutlin (GSE86164 [34],), revealed a strong peak within *ZNF185* promoter only in nutlin-treated cells (Fig 1B), suggesting p53 involvement in regulation of *ZNF185* transcription. Using previously described bioinformatic tool for p53 binding site (bs) prediction [35], we identified a putative binding site for p53 within the genomic region corresponding to the peak from the ChIP seq shown in Fig 1B (Fig 1C). Interestingly, this region is conserved only in primates and is absent in other species (Fig 1C). To confirm physical binding of p53 on *ZNF185* promoter, we performed ChIP assay in p53 Tet-On inducible SaOs-2 cells previously generated in the laboratory [36]. As a positive control, we used the promoter of *CDKN1A*, the gene coding for p21 (Fig 1D). To confirm if p53 could directly regulate *ZNF185* expression binding its promoter, we cloned the genomic locus harbouring p53 bs up-stream of the luciferase reporter gene. Luciferase activity assay showed a strong activation (120-fold, *P*<0.01) upon p53 overexpression. Interestingly, the overexpression of the two different p53 mutants frequently found in human cancers (R175H and R273H) did not show any strong activation compared to the control (Fig 1E), indicating that *ZNF185* is target of wild-type p53. Furthermore, the substitution of cytosines and guanines to adenines within *ZNF185* promoter sequence led to dramatic decrease of the luciferase activity (83% reduction, *P*<0.01) upon p53 overexpression (Fig 1F). To investigate if p53 is able to regulate *ZNF185* transcription, we induced p53 expression by doxycycline in SaOs-2 Tet-On cells and measured by RT-qPCR a significant increase of *ZNF185* mRNA after p53 induction paralleling *CDKN1A* increases (15-fold for *ZNF185* and 5-fold for *CDKN1A* at 24 h of induction, *P*<0.05; Fig 1G). We also confirmed this result changing cellular system. Indeed, overexpression of p53 in H1299 also led to a 20-fold increase of *ZNF185* mRNA, meanwhile the overexpression of two p53 mutants didn’t show any significant modulation (Fig 1H). Altogether, these data indicate *ZNF185* as a *bona fide* transcriptional target of wild-type p53.

### ZNF185 is up-regulated upon DNA damage

**Figure 2:**
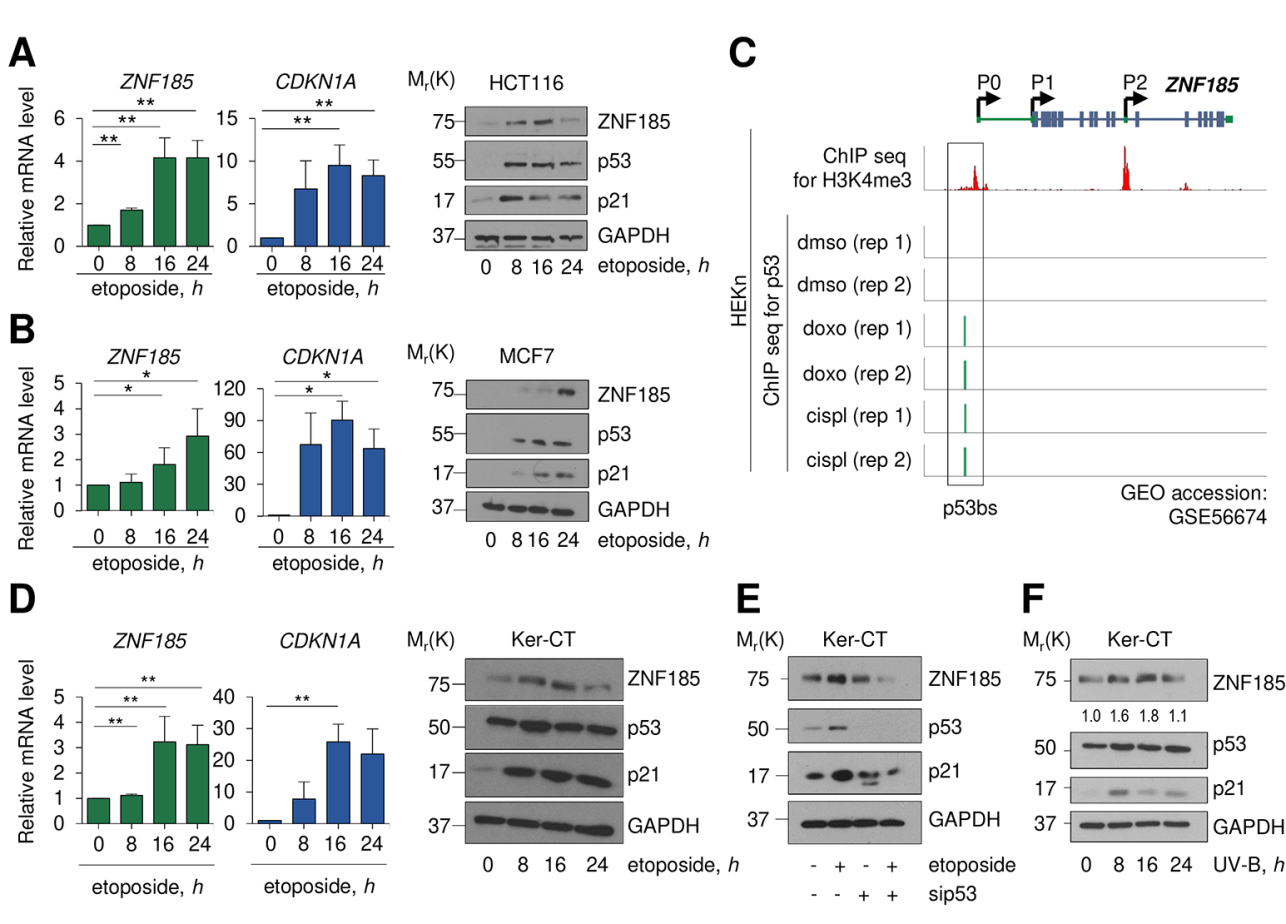
**ZNF185 is up-regulated upon DNA damage.** (**A**) qPCR analysis of *ZNF185* and *CDKN1A* mRNA levels in HCT116 after 25 µM etoposide treatment. **P<0.01. Western blot shows ZNF185, p53, and p21 levels. (**B**) qPCR analysis of *ZNF185* and *CDKN1A* mRNA levels in MCF7 after 25 µM etoposide treatment. Western blot shows ZNF185, p53, and p21 levels. *P<0.05 (**C**) Genomic locus of *ZNF185* showing the promoter region with H3K4me3 and p53 ChIP-seq signals after HEKn treatment with either DMSO, doxorubicin, or cisplatin. (**D**) qPCR analysis of *ZNF185* and *CDKN1A* mRNA levels in Ker-CT after 100 µM etoposide treatment. **P 0.05, *n*=3 (for *ZNF185*) and *n*=2 (for *CDKN1A*). Western blot shows ZNF185, p53, and p21 levels. (**E**) Western blot analysis of ZNF185, p53, and p21 levels after 100 µM etoposide treatment and p53 knock-down in Ker-CT cells. (**F**) Western blot analysis of ZNF185, p53, and p21 levels after 10 mJ^.^cm^-2^ UV-B treatment in Ker-CT cells for indicated times. Densitometry values of ZNF185 expression levels, normalized to GAPDH level, are shown.

We investigated whether *ZNF185* is transcribed as consequence of p53 activation following DNA damage. Using two different carcinoma cell lines harbouring wild-type p53 (HCT116 and MCF7), we analysed ZNF185 expression after 0, 8, 16, and 24 hours of etoposide treatment. In both cases, we saw p53 stabilization as indicated by the western blots (Fig 2 A-B) and, as a consequence of p53 activation, significant up-regulation of *ZNF185* mRNA (3-4-fold over control at 24 h of etoposide treatment, *P*<0.05), and p21 as positive control, both at mRNA and protein levels (Fig 2A-B). Interestingly, analysis of publicity available ChIP seq data (GSE56674 [37],) for p53 performed in keratinocytes showed that p53 binds to the locus within *ZNF185* promoter identified by us in this study. Moreover, this binding is observed only upon cisplatin or doxorubicin treatment (Fig 2C). As a model of basal layer keratinocytes, we used the commercial cell line of immortalized keratinocytes, Ker-CT. We confirmed that also in Ker-CT cells etoposide treatment leads to p53 stabilization and ZNF185 up-regulation both at mRNA (3-fold, *P*<0.01) and protein levels (Fig 2D). To confirm that ZNF185 up-regulation is p53-dependent, we performed siRNA-mediated knock-down of p53 in Ker-CT cells. As expected, depletion of p53 abolished up-regulation of ZNF185 upon etoposide treatment (Fig 2E). Since the major source of DNA damage in the human keratinocytes is UV irradiation, we irradiated Ker-CT cells and analysed ZNF185 level. Also in this case, we saw an up-regulation of ZNF185 at protein level. Altogether, these findings show that upon DNA damage we detected up-regulation of ZNF185 expression in p53-dependent manner both in tumour cell lines and in normal human keratinocytes.

### ZNF185 is involved in the cytoskeleton remodelling upon DNA damage

**Figure 3:**
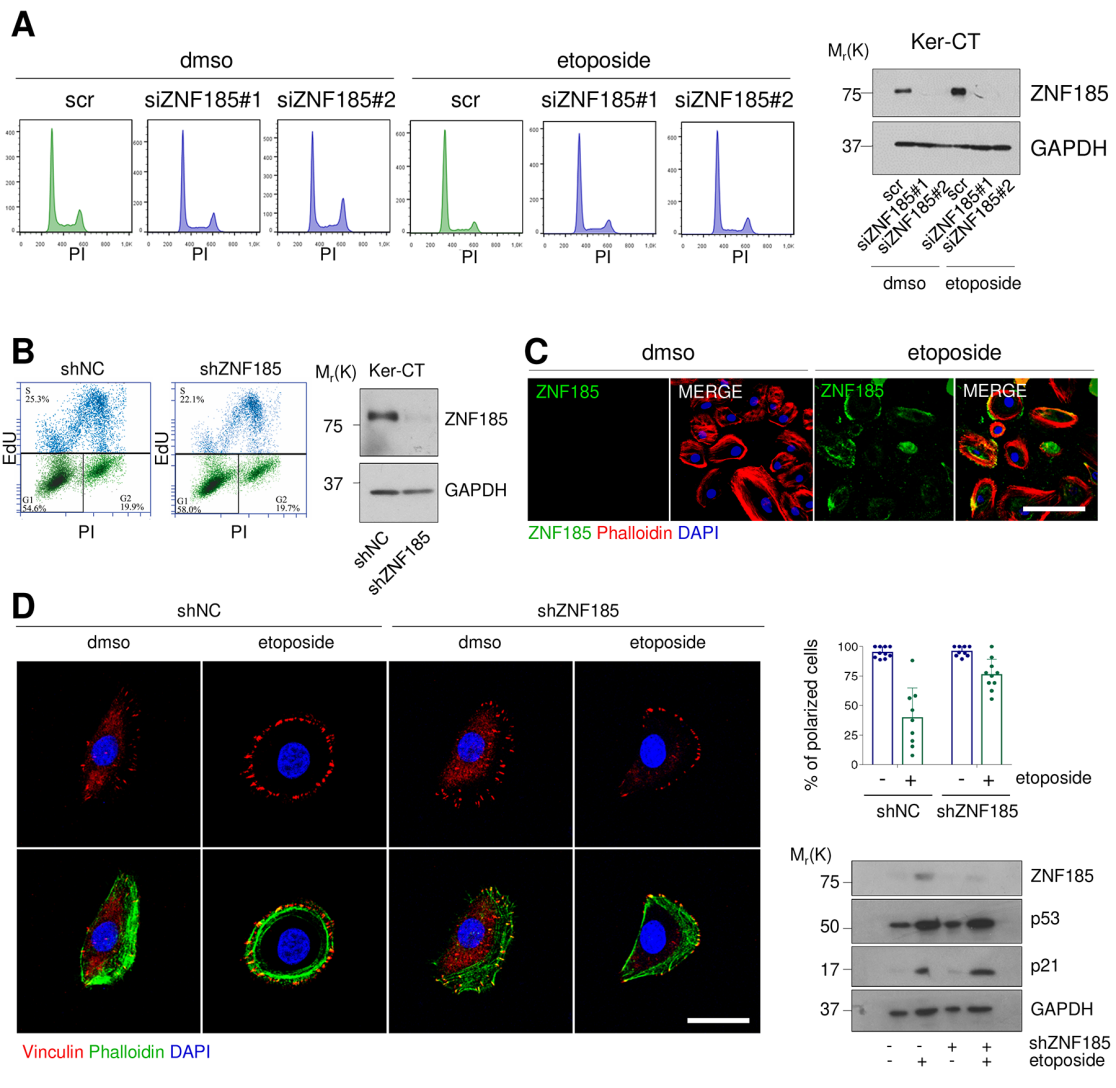
**ZNF185 is involved in the cytoskeleton remodelling upon DNA damage.** (**A**) FACS analysis of cell cycle content of the Ker-CT treated with either DMSO or 100 µM etoposide for 24 h after ZNF185 knock-down with two different siRNAs. Western blot confirms ZNF185 silencing. (**B**) EdU-incorporation assay by FACS showing % of EdU-positive Ker-CT shZNF185 cells. Western blot confirms the ZNF185 knock-down. (**C**) Immunofluorescence analysis of ZNF185 expression in Ker-CT treated with either DMSO or 100 µM etoposide for 16 h. Phalloidin was used for cytoskeleton staining. Scale bar: 50 µm. (**D**) Immunofluorescence analysis of vinculin distribution in Ker-CT treated with either DMSO or 100 µM etoposide and knocked-down for ZNF185. Phalloidin was used for cytoskeleton staining. Scale bar: 20 µm. In the right panel is shown the quantification of % of polarized cells in ten random fields. Western blot shows ZNF185, p53, and p21 levels.

Since the major functions of p53 activation upon DNA damage relate to the cell cycle arrest and apoptosis, we asked whether depletion of ZNF185 could alter cell cycle content under this specific stress condition. We performed siRNA mediated knock-down of ZNF185 in Ker-CT cells with two different siRNAs and treated the cells with etoposide. Cytofluorimetric analysis did not reveal any significant modulation in cell cycle distribution and apoptosis respect to the control (Fig 3A). It was previously reported that ZNF185 regulates proliferation of prostate cancer cells [38], to further investigate this point we generated Ker-CT cell line, stably expressing shRNA against ZNF185 (shZNF185). We performed the EdU-incorporation assay to evaluate the number of cells in S-phase, but we did not observe any significant difference in cell proliferation respect to the control (Fig 3B). Given that several LIM-domain Zn-fingers can migrate into the nucleus under stress conditions [39], we asked whether DNA damage can alter ZNF185 localisation. We found that ZNF185 localised in the cytoplasm and at the cell periphery (Fig 3C) also after etoposide treatment. Due to the presence of the actin-interacting domain within ZNF185 protein, we hypothesised that ZNF185 could be involved in cytoskeleton remodelling upon DNA damage. To this aim, we performed immunofluorescence analysis using phalloidin as a marker of filamentous actin and vinculin as a marker of focal adhesion. Under normal conditions, most of the cells had migratory phenotype showing vinculin accumulation on the leading edge. After etoposide treatment, cells lost planar polarity as visualised by homogeneous vinculin distribution on the cell periphery (percentage of polarized cells from 100% to 35%). Surprisingly, this phenotype was abolished in the shZNF185 cells which retained planar polarization also upon etoposide treatment (percentage of polarized cells from 100% to 82%) (Fig 3D). Altogether, these results suggest that ZNF185 is involved in the loss of the planar polarity of cells upon DNA damage.

### ZNF185 is down-regulated in epithelial cancers

**Figure 4:**
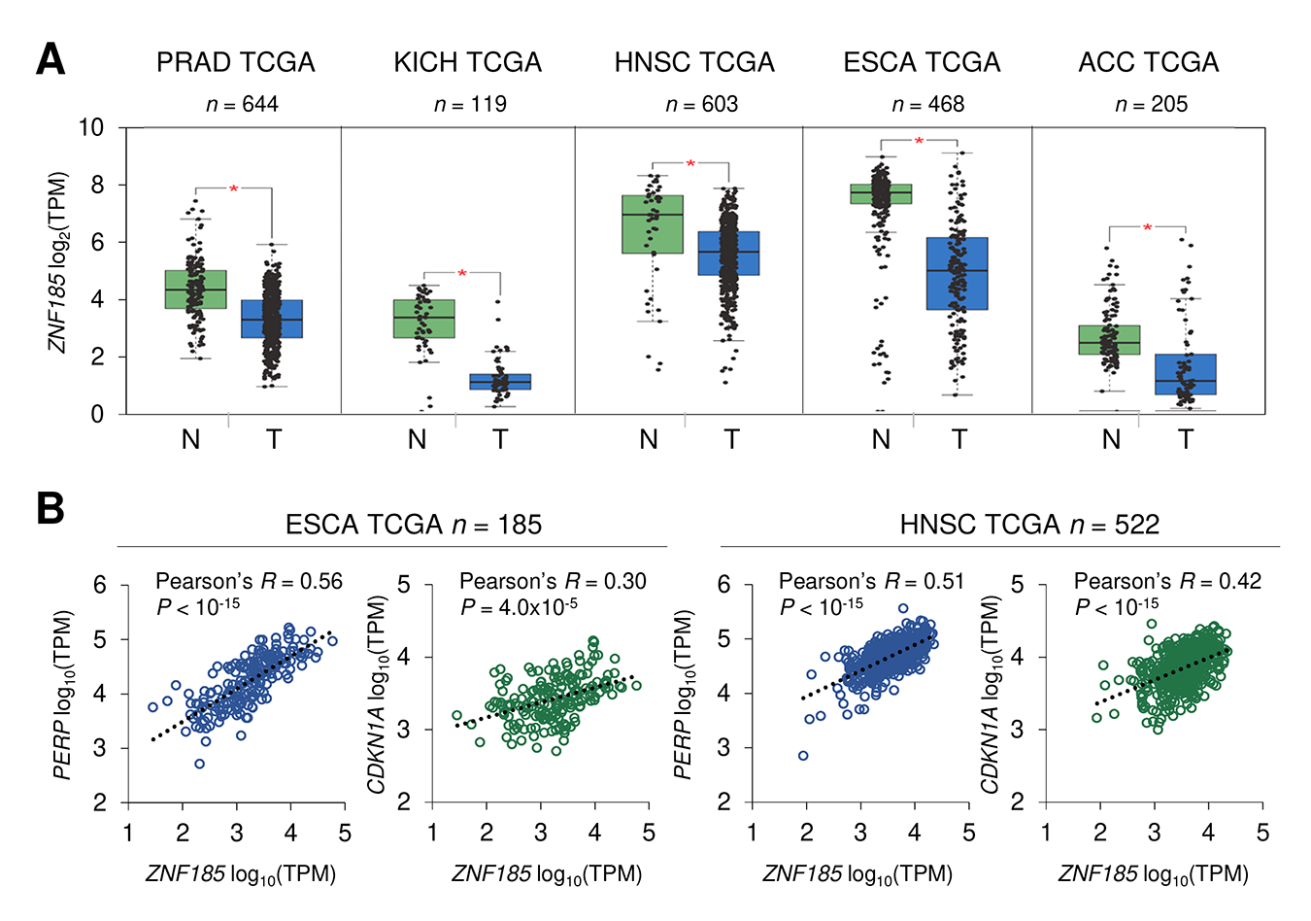
**ZNF185 mRNA is down-regulated in epithelial cancer.** (**A**) Box-plot showing expression of ZNF185 mRNA in different types of cancer from the GTEx/TCGA datasets for normal samples (N) and TCGA datasets for tumour samples (T). * *P*<0.05. (**B**) Correlation analysis between the expression of *ZNF185* and either *PERP* or *CDKN1A* in ESCA and HNSC tumour samples from TCGA.

**Figure 5:**
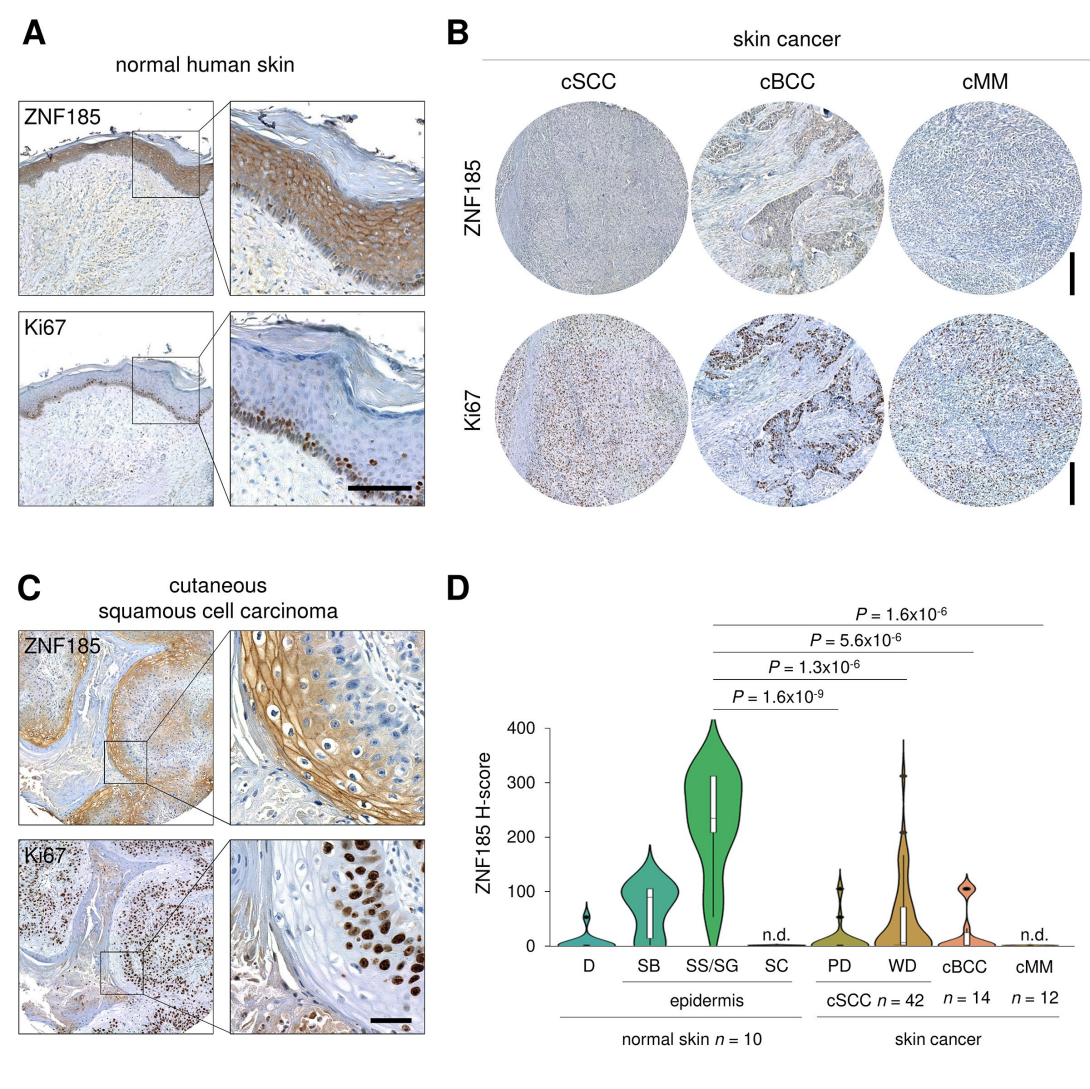
**ZNF185 is down-regulated in skin cancer.** (**A**) Immunohistochemical analysis of ZNF185 expression in normal skin. Ki67 was used as a marker of proliferation. Scale bar: 100 µm. (**B**) Immunohistochemical analysis of ZNF185 expression in skin cancer: cutaneous basal cell (cBCC), squamous cell carcinoma (cSCC), and malignant melanoma (cMM). Ki67 was used as a marker of proliferation. Scale bar: 250 µm. (**C**) Immunohistochemical analysis of ZNF185 expression in cutaneous squamous cell carcinoma (well differentiated). Ki67 was used as a marker of proliferation. Scale bar: 50 µm. (**D**) Violin plot showing H-score of protein expression level of ZNF185 in the normal skin (D – dermis, SB – basal layer, SS/SG – spinous and granular layers, SC – cornified layer) and skin cancer (cSCC (cSCC subpopulations: PD – poorly-differentiated cells, WD – well-differentiated cells), cBCC, and cMM).

p53 is frequently mutated in human cancers and we have shown that ZNF185 is positively regulated by wild-type p53, therefore we asked if ZNF185 level is decreased in epithelial cancers and particularly in the skin carcinomas. Firstly, we analysed *ZNF185* mRNA expression in different types of epithelial cancers from TCGA collection. Five cancer types - prostate adenocarcinoma (*PRAD*), chromophobe renal cell carcinoma (*KICH*), head and neck squamous cell carcinoma (*HNSC*), oesophageal carcinoma (*ESCA*), and adenoid cystic carcinoma (*ACC*) **–** showed a significant decrease in *ZNF185* mRNA level respect to the normal tissues from both TCGA and GTEx database (Fig 4A). Furthermore, we analysed correlation between the expression of *ZNF185* and two distinct targets of p53 – *PERP* and *CDKN1A*. Interestingly, a strong positive correlation was observed only in the cancers arising from squamous epithelia – oesophageal and head and neck carcinomas (Fig. 4B). Since there are only few datasets of skin cancer with a very low number of samples, we decided to analyse ZNF185 expression in skin cancer by immunohistochemistry using tissue microarray, containing 42 samples of the cutaneous squamous cell carcinoma (cSCC), 14 samples of the cutaneous basal cell carcinoma (cBCC), 12 samples of cutaneous malignant melanoma (cMM), and 10 samples of the normal skin. As a marker of proliferation, we used Ki67. Analysis of ZNF185 expression pattern at protein level in the normal skin confirmed previously published data from our laboratory [33], in which ZNF185 highest expression occurs in the differentiated spinous and granular layers (“SS/SG”) of the epidermis with low expression in the proliferating basal layer (“SB”). Cornified layer (“SC”) and dermis (“D”) were found negative for ZNF185 (Figure 5A). Analysis of skin cancer samples revealed that ZNF185 expression is dramatically down-regulated in the cutaneous squamous and basal cell carcinoma (“cSCC” and “cBCC”) and malignant melanoma (“cMM”) samples (Figure 5B). Furthermore, ZNF185 was found only in well-differentiated subpopulations of squamous cell carcinoma (“WD” of cSCC) in contrast to poorly-differentiated basal-like subpopulations (“PD” of cSCC) (Figure 5C). All the tumour samples showed a significant decrease (*P*<1x10^-5^) of ZNF185 H-score respect to the differentiated layers of the normal epidermis (Figure 5D). These findings reveal a dramatic down-regulation of ZNF185 at protein level in the skin cancer and suggest that ZNF185 could be a potential biomarker for epithelial cancer diagnosis and prognosis.

## DISCUSSION

Aging is complex set of genetic [40,41], epigenetic [42–46], immunological [47–51], and metabolic [52–58] rearrangements, involving several cellular signalling pathways [59–63] able to regulate metabolism, ROS formation [64–71] and DNA Damage Response (DDR) [72–74] in all organs [75–77]. Therefore, the identification of novel pathways involving p53-mediated responses [78,79] is of crucial interest. TP53 has been extensively studied in development and differentiation [14,80–82] as well as in cancer progression [83,84] and specifically in DDR [85–87]. The situation involves additional complexity if we consider that also the p53-related family members [88,89], that is p63 [90–92] and p73 [93], may be involved in DDR. TP63 is a gene primarily related to skin development [94], metabolism [36,95,96] and epithelial homeostasis [97,98] however, there is compelling evidence that it is also involved in cancer [27,47,99–103]. Similarly, TP73 is clearly involved in cancer [104], while the knockout studies [105,106] indicate a clear involvement in metabolism [107–109], neuro-development [104,110,111] and cancer progression [112,113]. Therefore, while the potential involvement of p73 with ZNF185 remains to be elucidated, the identification of a novel pathways of p53 regulating DDR via ZNF185 is of relevance.

Recently, the importance of actin-cytoskeleton remodelling and cell polarity during cancer cell spreading and metastasis has emerged [114–117], and p53 is in part involved in counteracting this specific aspect. Indeed, wild-type p53 can influence actin cytoskeleton dynamics controlling integrin and cadherin signalling and extracellular matrix degradation, suppressing EMT via different pathways [20,23,118–120]. Interestingly, also tumour microenvironment influences actin cytoskeleton [121,122], in part repressing wild-type p53 functions [123]. In fact, when p53 is inactivated, cancer cells invasion increases [124]. Here, we demonstrated that p53 wild-type transcriptionally activates *ZNF185* in cells upon DNA damage, which could make part of p53 negative regulation of cancer cell mobility, invasion and metastasis. Interestingly, similarly to another p53 target gene Rap2B [125], down-regulation of ZNF185 does not affect cell cycle progression or cell death, but its silencing abolishes the actin cytoskeleton rearrangements and cell polarity changes upon etoposide treatment.

ZNF185 is an actin-cytoskeleton-associated Lin-l 1, Isl-1 and Mec-3 (LIM) domain-containing protein [126]. The domain interacting with actin is located at the N-terminus, and it is necessary to mediate actin-cytoskeleton targeting of ZNF185, while the C-terminus LIM domain is dispensable for actin binding [38]. The LIM domain is a protein-protein interaction domain found in a wide range of proteins whose functions are related to the dynamics of the cytoskeleton [39,127,128]. In keratinocytes and epidermis ZNF185 has been described highly expressed in differentiating conditions, physically interacting with E-cadherin, a component of the adherens junctions, one of the critical cell-cell adhesive complexes crucial in the pluristratified epithelia [33]. ZNF185 involvement in pathologies, such as cancer [38], has not been completely investigated yet. Few studies reported ZNF185 as an unfavourable prognostic marker in ductal carcinoma of pancreas [129]. Its expression was found upregulated in colon cancer and likely correlated with liver metastasis [130]. On the other hand, other studies described epigenetic silencing of *ZNF185* associated with high grade and metastatic prostate tumours [131], lung tumours and head and neck squamous cell carcinomas [33,132–134]. Recently, it was reported that ZNF185 expression is negatively correlated with lymph node metastasis of lung adenocarcinoma and its overexpression leads to down-regulation of p-AKT, p-GSK3β, VEGF and MMP-9 expression [135]. These studies suggest a possible tumour-specific contribution of ZNF185 expression in tumour formation.

We confirmed ZNF185 down-regulation in different epithelial tumours and, by analysing the expression of ZNF185 at protein level, we found a significant decrease of ZNF185 in all the tumour samples analysed. Moreover, we found ZNF185 positive signal only in well-differentiated subpopulations of squamous cell carcinoma in contrast to poorly-differentiated basal-like aggressive subpopulations, suggesting a tumour-suppressor role of ZNF185. The possible involvement of ZNF185 in cytoskeleton remodelling upon DNA damage suggests its role in the metastasis promotion which is in line with previous reports [135]. The identification of p53-ZNF185 axis could contribute to determine how p53 controls cell spreading by actin cytoskeletal remodelling, in which both the mechanical properties of the cytoskeleton of the cell as well as the microenvironment of the tumour cells seem to play an important role. Further investigation on the mechanisms by which p53 controls actin cytoskeleton reorganization and cell polarity, including the identification of novel target genes and pathways, would possibly be useful in developing new anti-cancer strategies and therapies.

## MATERIALS AND METHODS

### Cell culture, transfection, and treatments

Immortalized human epidermal keratinocytes Ker-CT (ATCC, Manassas, VA, USA) were cultured in EpiLife medium with addition of Human Keratinocyte Growth Supplements (HKGS, LifeTechnologies, Carlsbad, CA, USA). Human Non-Small-Cell Lung Carcinoma cells H1299 (ATCC), Human Osteosarcoma cells SaOs-2 inducible for p53 expression (SaOs-2 Tet-on p53), Human Colorectal Carcinoma cells HCT116 (ATCC), and mammary gland adenocarcinoma cells MCF7 (ATCC) were grown in DMEM medium with the addition of 10% FBS, 100 U penicillin, and 100 μg/mL streptomycin (Gibco, LifeTechnologies). For siRNA-mediated knock-down experiments, 2.5x10^5^ of cells were seeded on 60 mm culture dishes and the day after transfected with 80 pmol of specific siRNAs (Supplementary Table 1) by Lipofectamine RNAiMAX transfection reagent (Invitrogen, Carlsbad, CA, USA). Cells were collected 48h posttransfection. For overexpression experiments, 1x10^6^ of cells were seeded in 100 mm culture dishes and the day after were transfected with 5 µg of plasmidic DNA using Lipofectamine 2000 transfection reagent (Invitrogen). Cells were collected 24 h later. For shRNA-mediated knock-down, 2.5x10^5^ of Ker-CT were infected at m.o.i. 10 with lentiviral particles carrying either scramble control shRNA (NC, Cat. No. VSC7078, Dharmacon, Lafayette, CO, USA) or specific shRNA for *ZNF185* (shZNF185, Cat. No. V3SVHSHC_5107270, Dharmacon). Transduced cells were selected by puromycin addiction to culture medium (1.5 µg/mL, Sigma, St. Louis, MO, USA). To induce DNA damage, cells were treated with either 25 µM or 100 µM etoposide (Sigma) or 0.02-0.40% DMSO (Sigma) for the indicated times or for 16 h if not otherwise indicated. Ker-CT cells were irradiated for indicated times with 10 mJ^.^cm^-2^ UV-B rays. p53 expression was induced in SaOs-2 Tet-On p53 cells by adding of 2 µg/mL doxycycline (Sigma) for the indicated times.

### Western blotting

The cells were collected by trypsinization, washed in PBS and lysed in RIPA buffer (50 mM Tris-cl pH 7.4, 150 mM NaCl, 1% NP40, 0.25% Na-deoxycholate, 1 mM AEBSF, 1 mM DTT). 20-50 µg of total protein extracts were resolved in SDS polyacrylamide gel using the Mini-PROTEAN Tetra cell System (Bio-Rad, Hercules, CA, USA) and blotted onto a Hybond PVDF membrane (GE Healthcare, Chicago, IL, USA) using the Bio-Rad Mini Trans-Blot Cell system Bio-Rad). Membranes were blocked with 5% non-fat dry milk (Bio-Rad) in PBS/0.1% Tween-20 buffer, for 1 h at room temperature in agitation. Membranes were incubated with primary antibodies over night at +4 °C, washed and hybridized for 1 h at room temperature with the appropriate horseradish peroxidase-conjugated secondary antibodies (goat anti-rabbit and goat anti-mouse antibodies, Bio-Rad). Detection was performed with the ECL chemiluminescence kit (Perkin Elmer, Waltham, MA, USA). The following antibodies were used: anti-ZNF185 (1:300, Cat. No. HPA000400, Sigma), anti-GAPDH (1:15000, Cat. No. G8795, Sigma), anti-p53 (1:500, Cat. No. SC-126, Santa Cruz, Dallas, TX, USA), anti-p21 (1:300, Cat. No. SC-756, Santa Cruz), anti-HA (1:1000, Cat. No. 901502, BioLegend, San Diego, CA, USA).

### RNA extraction and RT-qPCR analysis

Total RNA was isolated using the RNeasy Mini Kit (Qiagen, Hilden, Germany) following the manufacturer’s protocol. Total RNA (1 µg) was used for cDNA synthesis by GoScript Reverse Transcription System kit (Promega, Madison, WI, USA). RT-qPCRs were performed using the GoTaq Real-Time PCR System (Promega) in Applied Biosystems 7500 Real-Time PCR System (Applied Biosystems, Foster City, CA, USA) using appropriate qPCR primers (Supplementary Table 1). *TBP* was used as housekeeping gene for normalization. The expression of each gene was defined from the threshold cycle (C_t_), and relative expression levels were calculated using the 2^−ΔΔCt^ method. All reactions were run in triplicate.

### Analysis of *ZNF185* genomic locus

To analyse *ZNF185* genomic locus, different publicity accessible high-throughput sequencing data from ENCODE database (ChIP seq for H3K4me3 in different cell lines, CpG islands, DNase clusters, vertebrate conservation, TF binding) were visualised in the UCSC Genome Browser. Several ChIP-seq data from NCBI GEO database were analysed to assess p53 binding to the *ZNF185* promoter locus (GSE56674 ([37]) and GSE86164 ([34])). To identify putative p53 binding sites was used the “p53 scan” software [136]. The conservation analysis of *ZNF185* promoter locus was performed within UCSC genome browser.

### Chromatin immunoprecipitation assay

1x10^6^ of SaOs Tet-On p53 cells, induced to overexpress p53 for 16 h, were used for ChIP assay. Cells were collected, fixed in 1% formaldehyde, and subjected to sonication for DNA shearing. The chromatin immunoprecipitation was performed with HA antibody (BioLegend) or unspecific immunoglobulin G (IgG, Invitrogen) using the MAGnify ChIP Kit (Invitrogen). Specific primers were used to amplify the putative p53 response element identified within *ZNF185* promoter region (Supplementary Table 1).

### Luciferase activity assay

Promoter region of *ZNF185* containing the putative p53 binding site was amplified from human genomic DNA using specific primers (Supplementary Table 1). PCR products were digested by Kpn1/Nhe1 restriction enzymes (New England Biolabs, Ipswich, MA, USA) and subcloned into the pGL3-Promoter reporter vector (Promega). The constructs were completely sequenced. For luciferase activity assay, a total of 1.2x10^5^ H1299 cells were seeded in 12-well dishes 24 h before transfection. 100 ng of pGL3 reporter vector, 2 ng of pRL-CMV-Renilla luciferase vector (Promega) and 300 ng of either pcDNA-HA-p53, pcDNA-HA-p53-R175H, pcDNA-HA-p53-R273H, or empty pcDNA-HA vector (as a control) were cotransfected using Effectene transfection reagent according to the manufacturer’s instructions (Qiagen). The luciferase activity was measured 24 h after transfection using a Dual Luciferase Reporter Assay System (Promega). The light emission was measured over 10 sec using a Lumat LB9507 luminometer (EG&GBerthold, Bad Wildbad, Germany). The transfection efficiency was normalized to Renilla luciferase activity. The overexpression of p53 was confirmed by western blotting.

### Mutagenesis

For mutagenesis of p53 binding site was performed a PCR on 100 ng of pGL3 vector carrying p53 binding site using specific primers (Supplementary Table 1). PCR product was digested with DpnI restriction enzyme (New England Biolabs). The presence of mutated site was confirmed by sequencing.

### Immunohistochemical staining and TMA

The immunohistochemical staining of the skin cancer tissue microarray sections (US Biomax, Rockville, MD, USA) was performed using the BenchMark ULTRA slide staining system (Roche Diagnostics, Risch-Rotkreuz, Switzerland). The staining for Ki67 was performed using anti-Ki67 antibody (Cat. No. 790-4286, Ventana) following manufacturer’s indications. For ZNF185 staining, samples were incubated at 95°C for 76 min in the Cell Conditioning solution CC1 (Roche) and stained with anti-ZNF185 antibody (1:100, Sigma) for 40 min. Sections were counterstained with Mayer’s haematoxylin, dehydrated and mounted. Samples were scored using a semi-quantitative method. Cases were analysed for staining intensity, which was scored as 0 (not detected), 1+ (weak), 2+ (intermediate), and 3+ (strong). For each case, the histological “H-score” (0-300) was calculated by multiplying the percentage of positive cells (0%-100%) by the intensity (0-3).

### Immunofluorescence

Ker-CT cells were seeded on 5 mm coverslips, fixed for 10 min in 10% formalin buffered solution, washed with PBS and permeabilized in 0.2% triton X-100 solution in PBS for 10 min. Sections were incubated for 1 h in 10% goat serum in PBS at room temperature and overnight at 4 ºC with primary antibodies. Following antibodies were used: anti-ZNF185 (1:50, Sigma) and anti-vinculin (1:300, BD Biosciences, Franklin Lakes, NJ, USA). Sections were incubated for 1 h at room temperature with secondary anti-mouse and anti-rabbit 488- or 568-AlexaFluor conjugated antibodies (Invitrogen, 1:1000) together with 1 μg/mL DAPI (Sigma) for nuclear DNA staining. For cytoskeleton staining, 488- or 568-AlexaFluor conjugated phalloidin was used (1:1000, Thermo Fisher Scientific, Waltham, MA, USA). Images were acquired by Nikon A1 confocal laser microscope using NIS elements software (Nikon, Tokyo, Japan).

### Cell proliferation

The incorporation of EdU during DNA synthesis was evaluated using the Click-iT EdU flow cytometry assay kit according to the manufacturer's protocol (Thermo Fisher Scientific). The cell cycle was analysed using an Accuri C6 flow cytometer (BD Biosciences). Fifteen thousand events were evaluated using the Accuri C6 (BD Biosciences) software. For cell cycle analysis, cells were fixed in 50% methanol/acetone 4:1 mix for 30 min at +4 °C, then treated with 13 Kunitz U/mL RNase for 15 min and stained with 50 µg/mL of propidium iodide for 20 min. Twelve thousand events were acquired using FACScalibur (BD Biosciences). Cell cycle distribution was calculated using FloxJo software.

### Bioinformatic analysis

Analysis of ZNF185 expression in normal and tumour samples from TCGA/GTEx databases was performed using GEPIA [137]. *ZNF185*, *PERP*, and *CDKN1A* expression data in ESCA and HNSC samples from TCGA collection were obtained using R2: Genomics Analysis and Visualization Platform (http://r2.amc.nl/).

### Statistical analysis

The significance of differences between two experimental groups was calculated using unpaired, two-tailed Student’s t-test. Values of *P* < 0.05 were considered significant. For RT-qPCR and luciferase assay, values reported are the mean ± SD. For statistical analysis of TMA scoring was used Mann–Whitney U test. All statistical analyses were performed using GraphPad Prism 7.0 Software. Violin plots were generated in R using ggplot2 package.

## SUPPLEMENTARY MATERIAL

Supplementary Table 1

## References

[1] Harper JW, Elledge SJ. The DNA damage response: ten years after. Mol Cell. 2007; 28:739–45. 10.1016/j.molcel.2007.11.015 18082599

[2] Jackson SP, Bartek J. The DNA-damage response in human biology and disease. Nature. 2009; 461:1071–78. 10.1038/nature08467 19847258 PMC2906700

[3] Houtgraaf JH, Versmissen J, van der Giessen WJ. A concise review of DNA damage checkpoints and repair in mammalian cells. Cardiovasc Revasc Med. 2006; 7:165–72. 10.1016/j.carrev.2006.02.002 16945824

[4] Ciccia A, Elledge SJ. The DNA damage response: making it safe to play with knives. Mol Cell. 2010; 40:179–204. 10.1016/j.molcel.2010.09.019 20965415 PMC2988877

[5] Hawley BR, Lu WT, Wilczynska A, Bushell M. The emerging role of RNAs in DNA damage repair. Cell Death Differ. 2017; 24:1989. 10.1038/cdd.2017.146 28862702 PMC5635227

[6] Baran K, Yang M, Dillon CP, Samson LL, Green DR. The proline rich domain of p53 is dispensable for MGMT-dependent DNA repair and cell survival following alkylation damage. Cell Death Differ. 2017; 24:1925–36. 10.1038/cdd.2017.116 28753207 PMC5635218

[7] Delbridge AR, Grabow S, Strasser A. Loss of BIM augments resistance of ATM-deficient thymocytes to DNA damage-induced apoptosis but does not accelerate lymphoma development. Cell Death Differ. 2017; 24:1987–88. 10.1038/cdd.2017.138 28885618 PMC5635225

[8] Zhou BB, Elledge SJ. The DNA damage response: putting checkpoints in perspective. Nature. 2000; 408:433–39. 10.1038/35044005 11100718

[9] Lakin ND, Jackson SP. Regulation of p53 in response to DNA damage. Oncogene. 1999; 18:7644–55. 10.1038/sj.onc.1203015 10618704

[10] Kruiswijk F, Labuschagne CF, Vousden KH. p53 in survival, death and metabolic health: a lifeguard with a licence to kill. Nat Rev Mol Cell Biol. 2015; 16:393–405. 10.1038/nrm4007 26122615

[11] Kubbutat MH, Jones SN, Vousden KH. Regulation of p53 stability by Mdm2. Nature. 1997; 387:299–303. 10.1038/387299a0 9153396

[12] Gurley KE, Ashley AK, Moser RD, Kemp CJ. Synergy between Prkdc and Trp53 regulates stem cell proliferation and GI-ARS after irradiation. Cell Death Differ. 2017; 24:1853–60. 10.1038/cdd.2017.107 28686579 PMC5635213

[13] Nayak D, Kumar A, Chakraborty S, Rasool RU, Amin H, Katoch A, Gopinath V, Mahajan V, Zilla MK, Rah B, Gandhi SG, Ali A, Kumar LD, Goswami A. Inhibition of Twist1-mediated invasion by Chk2 promotes premature senescence in p53-defective cancer cells. Cell Death Differ. 2017; 24:1275–87. 10.1038/cdd.2017.70 28498365 PMC5520175

[14] Van Nostrand JL, Bowen ME, Vogel H, Barna M, Attardi LD. The p53 family members have distinct roles during mammalian embryonic development. Cell Death Differ. 2017; 24:575–79. 10.1038/cdd.2016.128 28211873 PMC5384018

[15] Solomon H, Bräuning B, Fainer I, Ben-Nissan G, Rabani S, Goldfinger N, Moscovitz O, Shakked Z, Rotter V, Sharon M. Post-translational regulation of p53 function through 20S proteasome-mediated cleavage. Cell Death Differ. 2017; 24:2187–98. 10.1038/cdd.2017.139 28885617 PMC5686354

[16] Riley T, Sontag E, Chen P, Levine A. Transcriptional control of human p53-regulated genes. Nat Rev Mol Cell Biol. 2008; 9:402–12. 10.1038/nrm2395 18431400

[17] Ford JM. Regulation of DNA damage recognition and nucleotide excision repair: another role for p53. Mutat Res. 2005; 577:195–202. 10.1016/j.mrfmmm.2005.04.005 15927209

[18] Ebata T, Hirata H, Kawauchi K. Functions of the Tumor Suppressors p53 and Rb in Actin Cytoskeleton Remodeling. BioMed Res Int. 2016; 2016:9231057. 10.1155/2016/9231057 28078303 PMC5203884

[19] Roger L, Gadea G, Roux P. Control of cell migration: a tumour suppressor function for p53? Biol Cell. 2006; 98:141–52. 10.1042/BC20050058 16480340

[20] Araki K, Ebata T, Guo AK, Tobiume K, Wolf SJ, Kawauchi K. p53 regulates cytoskeleton remodeling to suppress tumor progression. Cell Mol Life Sci. 2015; 72:4077–94. 10.1007/s00018-015-1989-9 26206378 PMC11114009

[21] Janouskova H, Maglott A, Leger DY, Bossert C, Noulet F, Guerin E, Guenot D, Pinel S, Chastagner P, Plenat F, Entz-Werle N, Lehmann-Che J, Godet J, et al. Integrin α5β1 plays a critical role in resistance to temozolomide by interfering with the p53 pathway in high-grade glioma. Cancer Res. 2012; 72:3463–70. 10.1158/0008-5472.CAN-11-4199 22593187

[22] Janouskova H, Ray AM, Noulet F, Lelong-Rebel I, Choulier L, Schaffner F, Lehmann M, Martin S, Teisinger J, Dontenwill M. Activation of p53 pathway by Nutlin-3a inhibits the expression of the therapeutic target α5 integrin in colon cancer cells. Cancer Lett. 2013; 336:307–18. 10.1016/j.canlet.2013.03.018 23523610

[23] Bon G, Di Carlo SE, Folgiero V, Avetrani P, Lazzari C, D’Orazi G, Brizzi MF, Sacchi A, Soddu S, Blandino G, Mottolese M, Falcioni R. Negative regulation of beta4 integrin transcription by homeodomain-interacting protein kinase 2 and p53 impairs tumor progression. Cancer Res. 2009; 69:5978–86. 10.1158/0008-5472.CAN-09-0244 19567674

[24] Muller PA, Vousden KH, Norman JC. p53 and its mutants in tumor cell migration and invasion. J Cell Biol. 2011; 192:209–18. 10.1083/jcb.201009059 21263025 PMC3172183

[25] Gadea G, de Toledo M, Anguille C, Roux P. Loss of p53 promotes RhoA-ROCK-dependent cell migration and invasion in 3D matrices. J Cell Biol. 2007; 178:23–30. 10.1083/jcb.200701120 17606864 PMC2064414

[26] Lefort K, Mandinova A, Ostano P, Kolev V, Calpini V, Kolfschoten I, Devgan V, Lieb J, Raffoul W, Hohl D, Neel V, Garlick J, Chiorino G, Dotto GP. Notch1 is a p53 target gene involved in human keratinocyte tumor suppression through negative regulation of ROCK1/2 and MRCKalpha kinases. Genes Dev. 2007; 21:562–77. 10.1101/gad.1484707 17344417 PMC1820898

[27] Guo F, Gao Y, Wang L, Zheng Y. p19Arf-p53 tumor suppressor pathway regulates cell motility by suppression of phosphoinositide 3-kinase and Rac1 GTPase activities. J Biol Chem. 2003; 278:14414–19. 10.1074/jbc.M300341200 12578823

[28] Gadéa G, Lapasset L, Gauthier-Rouvière C, Roux P. Regulation of Cdc42-mediated morphological effects: a novel function for p53. EMBO J. 2002; 21:2373–82. 10.1093/emboj/21.10.2373 12006490 PMC126005

[29] Croft DR, Crighton D, Samuel MS, Lourenco FC, Munro J, Wood J, Bensaad K, Vousden KH, Sansom OJ, Ryan KM, Olson MF. p53-mediated transcriptional regulation and activation of the actin cytoskeleton regulatory RhoC to LIMK2 signaling pathway promotes cell survival. Cell Res. 2011; 21:666–82. 10.1038/cr.2010.154 21079653 PMC3145139

[30] Ongusaha PP, Kim HG, Boswell SA, Ridley AJ, Der CJ, Dotto GP, Kim YB, Aaronson SA, Lee SW. RhoE is a pro-survival p53 target gene that inhibits ROCK I-mediated apoptosis in response to genotoxic stress. Curr Biol. 2006; 16:2466–72. 10.1016/j.cub.2006.10.056 17174923 PMC2779528

[31] Hall AE, Lu WT, Godfrey JD, Antonov AV, Paicu C, Moxon S, Dalmay T, Wilczynska A, Muller PA, Bushell M. The cytoskeleton adaptor protein ankyrin-1 is upregulated by p53 following DNA damage and alters cell migration. Cell Death Dis. 2016; 7:e2184. 10.1038/cddis.2016.91 27054339 PMC4855670

[32] Muller PA, Caswell PT, Doyle B, Iwanicki MP, Tan EH, Karim S, Lukashchuk N, Gillespie DA, Ludwig RL, Gosselin P, Cromer A, Brugge JS, Sansom OJ, et al. Mutant p53 drives invasion by promoting integrin recycling. Cell. 2009; 139:1327–41. 10.1016/j.cell.2009.11.026 20064378

[33] Smirnov A, Lena AM, Cappello A, Panatta E, Anemona L, Bischetti S, Annicchiarico-Petruzzelli M, Mauriello A, Melino G, Candi E. ZNF185 is a p63 target gene critical for epidermal differentiation and squamous cell carcinoma development. Oncogene. Epub ahead of print. 10.1038/s41388-018-0509-4 30337687 PMC6755960

[34] Andrysik Z, Galbraith MD, Guarnieri AL, Zaccara S, Sullivan KD, Pandey A, MacBeth M, Inga A, Espinosa JM. Identification of a core TP53 transcriptional program with highly distributed tumor suppressive activity. Genome Res. 2017; 27:1645–57. 10.1101/gr.220533.117 28904012 PMC5630028

[35] Kouwenhoven EN, van Heeringen SJ, Tena JJ, Oti M, Dutilh BE, Alonso ME, de la Calle-Mustienes E, Smeenk L, Rinne T, Parsaulian L, Bolat E, Jurgelenaite R, Huynen MA, et al. Genome-wide profiling of p63 DNA-binding sites identifies an element that regulates gene expression during limb development in the 7q21 SHFM1 locus. PLoS Genet. 2010; 6:e1001065. 10.1371/journal.pgen.1001065 20808887 PMC2924305

[36] Gressner O, Schilling T, Lorenz K, Schulze Schleithoff E, Koch A, Schulze-Bergkamen H, Lena AM, Candi E, Terrinoni A, Catani MV, Oren M, Melino G, Krammer PH, et al. TAp63α induces apoptosis by activating signaling via death receptors and mitochondria. EMBO J. 2005; 24:2458–71. 10.1038/sj.emboj.7600708 15944736 PMC1173149

[37] McDade SS, Patel D, Moran M, Campbell J, Fenwick K, Kozarewa I, Orr NJ, Lord CJ, Ashworth AA, McCance DJ. Genome-wide characterization reveals complex interplay between TP53 and TP63 in response to genotoxic stress. Nucleic Acids Res. 2014; 42:6270–85. 10.1093/nar/gku299 24823795 PMC4041465

[38] Zhang JS, Gong A, Young CY. ZNF185, an actin-cytoskeleton-associated growth inhibitory LIM protein in prostate cancer. Oncogene. 2007; 26:111–22. 10.1038/sj.onc.1209769 16799630

[39] Kadrmas JL, Beckerle MC. The LIM domain: from the cytoskeleton to the nucleus. Nat Rev Mol Cell Biol. 2004; 5:920–31. 10.1038/nrm1499 15520811

[40] Stoll EA, Karapavlovic N, Rosa H, Woodmass M, Rygiel K, White K, Turnbull DM, Faulkes CG. Naked mole-rats maintain healthy skeletal muscle and Complex IV mitochondrial enzyme function into old age. Aging (Albany NY). 2016; 8:3468–85. 10.18632/aging.101140 27997359 PMC5270680

[41] Ticinesi A, Tana C, Nouvenne A, Prati B, Lauretani F, Meschi T. Gut microbiota, cognitive frailty and dementia in older individuals: a systematic review. Clin Interv Aging. 2018; 13:1497–511. 10.2147/CIA.S139163 30214170 PMC6120508

[42] Kaestner L, Minetti G. The potential of erythrocytes as cellular aging models. Cell Death Differ. 2017; 24:1475–77. 10.1038/cdd.2017.100 28622292 PMC5563981

[43] Kuro-O M. Molecular Mechanisms Underlying Accelerated Aging by Defects in the FGF23-Klotho System. Int J Nephrol. 2018; 2018:9679841. 10.1155/2018/9679841 29951315 PMC5987335

[44] Pollard AK, Ortori CA, Stöger R, Barrett DA, Chakrabarti L. Mouse mitochondrial lipid composition is defined by age in brain and muscle. Aging (Albany NY). 2017; 9:986–98. 10.18632/aging.101204 28325886 PMC5391243

[45] Regina C, Compagnone M, Peschiaroli A, Lena AM, Melino G, Candi E. ΔNp63α modulates histone methyl transferase SETDB1 to transcriptionally repress target genes in cancers. Cell Death Dis. 2016; 2:16015. 10.1038/cddiscovery.2016.15 27551509 PMC4979509

[46] Smirnov A, Panatta E, Lena A, Castiglia D, Di Daniele N, Melino G, Candi E. FOXM1 regulates proliferation, senescence and oxidative stress in keratinocytes and cancer cells. Aging (Albany NY). 2016; 8:1384–97. 10.18632/aging.100988 27385468 PMC4993337

[47] Xu-Monette ZY, Zhang S, Li X, Manyam GC, Wang XX, Xia Y, Visco C, Tzankov A, Zhang L, Montes-Moreno S, Dybkaer K, Chiu A, Orazi A, et al. p63 expression confers significantly better survival outcomes in high-risk diffuse large B-cell lymphoma and demonstrates p53-like and p53-independent tumor suppressor function. Aging (Albany NY). 2016; 8:345–65. 10.18632/aging.100898 26878872 PMC4789587

[48] Lareau CA, DeWeese CF, Adrianto I, Lessard CJ, Gaffney PM, Iannuzzi MC, Rybicki BA, Levin AM, Montgomery CG. Polygenic risk assessment reveals pleiotropy between sarcoidosis and inflammatory disorders in the context of genetic ancestry. Genes Immun. 2017; 18:88–94. 10.1038/gene.2017.3 28275240 PMC5407914

[49] Messemaker TC, Mikkers HM, Huizinga TW, Toes RE, van der Helm-van Mil AH, Kurreeman F. Inflammatory genes TNFα and IL6 display no signs of increased H3K4me3 in circulating monocytes from untreated rheumatoid arthritis patients. Genes Immun. 2017; 18:191–96. 10.1038/gene.2017.20 28794503

[50] Nikolic T, Woittiez NJ, van der Slik A, Laban S, Joosten A, Gysemans C, Mathieu C, Zwaginga JJ, Koeleman B, Roep BO. Differential transcriptome of tolerogenic versus inflammatory dendritic cells points to modulated T1D genetic risk and enriched immune regulation. Genes Immun. 2017; 18:176–83. 10.1038/gene.2017.18 28794505

[51] Liu S, Hou XL, Sui WG, Lu QJ, Hu YL, Dai Y. Direct measurement of B-cell receptor repertoire’s composition and variation in systemic lupus erythematosus. Genes Immun. 2017; 18:22–27. 10.1038/gene.2016.45 28053320

[52] Uzhachenko R, Boyd K, Olivares-Villagomez D, Zhu Y, Goodwin JS, Rana T, Shanker A, Tan WJ, Bondar T, Medzhitov R, Ivanova AV. Mitochondrial protein Fus1/Tusc2 in premature aging and age-related pathologies: critical roles of calcium and energy homeostasis. Aging (Albany NY). 2017; 9:627–49. 10.18632/aging.101213 28351997 PMC5391223

[53] Baraibar MA, Hyzewicz J, Rogowska-Wrzesinska A, Bulteau AL, Prip-Buus C, Butler-Browne G, Friguet B. Impaired energy metabolism of senescent muscle satellite cells is associated with oxidative modifications of glycolytic enzymes. Aging (Albany NY). 2016; 8:3375–89. 10.18632/aging.101126 27922824 PMC5270674

[54] Honrath B, Matschke L, Meyer T, Magerhans L, Perocchi F, Ganjam GK, Zischka H, Krasel C, Gerding A, Bakker BM, Bünemann M, Strack S, Decher N, et al. SK2 channels regulate mitochondrial respiration and mitochondrial Ca^2+^ uptake. Cell Death Differ. 2017; 24:761–73. 10.1038/cdd.2017.2 28282037 PMC5423111

[55] Ingram T, Chakrabarti L. Proteomic profiling of mitochondria: what does it tell us about the ageing brain? Aging (Albany NY). 2016; 8:3161–79. 10.18632/aging.101131 27992860 PMC5270661

[56] Kagan VE, Jiang J, Huang Z, Tyurina YY, Desbourdes C, Cottet-Rousselle C, Dar HH, Verma M, Tyurin VA, Kapralov AA, Cheikhi A, Mao G, Stolz D, et al. NDPK-D (NM23-H4)-mediated externalization of cardiolipin enables elimination of depolarized mitochondria by mitophagy. Cell Death Differ. 2016; 23:1140–51. 10.1038/cdd.2015.160 26742431 PMC4946882

[57] Kaufman DM, Wu X, Scott BA, Itani OA, Van Gilst MR, Bruce JE, Crowder CM. Ageing and hypoxia cause protein aggregation in mitochondria. Cell Death Differ. 2017; 24:1730–38. 10.1038/cdd.2017.101 28644434 PMC5596417

[58] Giacomello M, Pellegrini L. The coming of age of the mitochondria-ER contact: a matter of thickness. Cell Death Differ. 2016; 23:1417–27. 10.1038/cdd.2016.52 27341186 PMC5072433

[59] Choi JY, Hwang CY, Lee B, Lee SM, Bahn YJ, Lee KP, Kang M, Kim YS, Woo SH, Lim JY, Kim E, Kwon KS. Age-associated repression of type 1 inositol 1, 4, 5-triphosphate receptor impairs muscle regeneration. Aging (Albany NY). 2016; 8:2062–80. 10.18632/aging.101039 27658230 PMC5076452

[60] Jęśko H, Stępień A, Lukiw WJ, Strosznajder RP. The Cross-Talk Between Sphingolipids and Insulin-Like Growth Factor Signaling: Significance for Aging and Neurodegeneration. Mol Neurobiol. 2018. 10.1007/s12035-018-1286-3 30140974 PMC6476865

[61] Lee SJ, Hwang J, Jeong HJ, Yoo M, Go GY, Lee JR, Leem YE, Park JW, Seo DW, Kim YK, Hahn MJ, Han JW, Kang JS, Bae GU. PKN2 and Cdo interact to activate AKT and promote myoblast differentiation. Cell Death Dis. 2016; 7:e2431. 10.1038/cddis.2016.296 27763641 PMC5133968

[62] Oh J, Sinha I, Tan KY, Rosner B, Dreyfuss JM, Gjata O, Tran P, Shoelson SE, Wagers AJ. Age-associated NF-κB signaling in myofibers alters the satellite cell niche and re-strains muscle stem cell function. Aging (Albany NY). 2016; 8:2871–96. 10.18632/aging.101098 27852976 PMC5191876

[63] Shao AW, Sun H, Geng Y, Peng Q, Wang P, Chen J, Xiong T, Cao R, Tang J. Bclaf1 is an important NF-κB signaling transducer and C/EBPβ regulator in DNA damage-induced senescence. Cell Death Differ. 2016; 23:865–75. 10.1038/cdd.2015.150 26794446 PMC4832105

[64] Knupp J, Martinez-Montañés F, Van Den Bergh F, Cottier S, Schneiter R, Beard D, Chang A. Sphingolipid accumulation causes mitochondrial dysregulation and cell death. Cell Death Differ. 2017; 24:2044–53. 10.1038/cdd.2017.128 28800132 PMC5686345

[65] Lalia AZ, Dasari S, Robinson MM, Abid H, Morse DM, Klaus KA, Lanza IR. Influence of omega-3 fatty acids on skeletal muscle protein metabolism and mitochondrial bioenergetics in older adults. Aging (Albany NY). 2017; 9:1096–129. 10.18632/aging.101210 28379838 PMC5425117

[66] Lang A, Anand R, Altinoluk-Hambüchen S, Ezzahoini H, Stefanski A, Iram A, Bergmann L, Urbach J, Böhler P, Hänsel J, Franke M, Stühler K, Krutmann J, et al. SIRT4 interacts with OPA1 and regulates mitochondrial quality control and mitophagy. Aging (Albany NY). 2017; 9:2163–89. 10.18632/aging.101307 29081403 PMC5680561

[67] Marini A, Rotblat B, Sbarrato T Niklison-Chirou MV, Knight JR, Dudek K, Jones C, Bushell M, Knight RA, Amelio I, Willis AE, Melino G. TAp73 contributes to the oxidative stress response by regulating protein synthesis. Proc Natl Acad Sci USA. 2018; 115:6219–24. 10.1073/pnas.1718531115 29844156 PMC6004440

[68] Pinto M, Pickrell AM, Wang X, Bacman SR, Yu A, Hida A, Dillon LM, Morton PD, Malek TR, Williams SL, Moraes CT. Transient mitochondrial DNA double strand breaks in mice cause accelerated aging phenotypes in a ROS-dependent but p53/p21-independent manner. Cell Death Differ. 2017; 24:288–99. 10.1038/cdd.2016.123 27911443 PMC5299712

[69] Qi Y, Liu H, Daniels MP, Zhang G, Xu H. Loss of Drosophila i-AAA protease, dYME1L, causes abnormal mitochondria and apoptotic degeneration. Cell Death Differ. 2016; 23:291–302. 10.1038/cdd.2015.94 26160069 PMC4716308

[70] Ohkusu-Tsukada K, Yamashita T, Tsukada T, Takahashi K. Low expression of a D^dm7^/L^dm7^-hybrid mutant (D/L^dm7^) in the novel haplotype H-2^nc^ identified in atopic dermatitis model NC/Nga mice. Genes Immun. 2017. 10.1038/s41435-017-0003-y 29282355

[71] Tak H, Eun JW, Kim J, Park SJ, Kim C, Ji E, Lee H, Kang H, Cho DH, Lee K, Kim W, Nam SW, Lee EK. T-cell-restricted intracellular antigen 1 facilitates mitochondrial fragmentation by enhancing the expression of mitochondrial fission factor. Cell Death Differ. 2017; 24:49–58. 10.1038/cdd.2016.90 27612012 PMC5260506

[72] Galluzzi L, Vitale I, Aaronson SA, Abrams JM, Adam D, Agostinis P, Alnemri ES, Altucci L, Amelio I, Andrews DW, Annicchiarico-Petruzzelli M, Antonov AV, Arama E, et al. Molecular mechanisms of cell death: recommendations of the Nomenclature Committee on Cell Death 2018. Cell Death Differ. 2018; 25:486–541. 10.1038/s41418-017-0012-4 29362479 PMC5864239

[73] Gross A, Zaltsman Y, Maryanovich M. The ATM-BID pathway plays a critical role in the DNA damage response by regulating mitochondria metabolism. Cell Death Differ. 2016; 23:182. 10.1038/cdd.2015.154 26611459 PMC4815972

[74] Lovat PE, Ranalli M, Annichiarrico-Petruzzelli M, Bernassola F, Piacentini M, Malcolm AJ, Pearson AD, Melino G, Redfern CP. Effector mechanisms of fenretinide-induced apoptosis in neuroblastoma. Exp Cell Res. 2000; 260:50–60. 10.1006/excr.2000.4988 11010810

[75] Belle JI, Petrov JC, Langlais D, Robert F, Cencic R, Shen S, Pelletier J, Gros P, Nijnik A. Repression of p53-target gene Bbc3/PUMA by MYSM1 is essential for the survival of hematopoietic multipotent progenitors and contributes to stem cell maintenance. Cell Death Differ. 2016; 23:759–75. 10.1038/cdd.2015.140 26768662 PMC4832099

[76] Brzeszczyńska J, Johns N, Schilb A, Degen S, Degen M, Langen R, Schols A, Glass DJ, Roubenoff R, Greig CA, Jacobi C, Fearon KC, Ross JA. Loss of oxidative defense and potential blockade of satellite cell maturation in the skeletal muscle of patients with cancer but not in the healthy elderly. Aging (Albany NY). 2016; 8:1690–702. 10.18632/aging.101006 27454226 PMC5032690

[77] Greschik H, Duteil D, Messaddeq N, Willmann D, Arrigoni L, Sum M, Jung M, Metzger D, Manke T, Günther T, Schüle R. The histone code reader Spin1 controls skeletal muscle development. Cell Death Dis. 2017; 8:e3173. 10.1038/cddis.2017.468 29168801 PMC5775400

[78] Aggarwal M, Saxena R, Sinclair E, Fu Y, Jacobs A, Dyba M, Wang X, Cruz I, Berry D, Kallakury B, Mueller SC, Agostino SD, Blandino G, et al. Reactivation of mutant p53 by a dietary-related compound phenethyl isothiocyanate inhibits tumor growth. Cell Death Differ. 2016; 23:1615–27. 10.1038/cdd.2016.48 27258787 PMC5041190

[79] Alexandrova EM, Moll UM. Depleting stabilized GOF mutant p53 proteins by inhibiting molecular folding chaperones: a new promise in cancer therapy. Cell Death Differ. 2017; 24:3–5. 10.1038/cdd.2016.145 27935583 PMC5260503

[80] Artigas N, Gámez B, Cubillos-Rojas M, Sánchez-de Diego C, Valer JA, Pons G, Rosa JL, Ventura F. p53 inhibits SP7/Osterix activity in the transcriptional program of osteoblast differentiation. Cell Death Differ. 2017; 24:2022–31. 10.1038/cdd.2017.113 28777372 PMC5686339

[81] Baldelli S, Ciriolo MR. Altered S-nitrosylation of p53 is responsible for impaired antioxidant response in skeletal muscle during aging. Aging (Albany NY). 2016; 8:3450–67. 10.18632/aging.101139 28025407 PMC5270679

[82] Cam H, Griesmann H, Beitzinger M, Hofmann L, Beinoraviciute-Kellner R, Sauer M, Hüttinger-Kirchhof N, Oswald C, Friedl P, Gattenlöhner S, Burek C, Rosenwald A, Stiewe T. p53 family members in myogenic differentiation and rhabdomyosarcoma development. Cancer Cell. 2006; 10:281–93. 10.1016/j.ccr.2006.08.024 17045206

[83] Ghosh S, Salot S, Sengupta S, Navalkar A, Ghosh D, Jacob R, Das S, Kumar R, Jha NN, Sahay S, Mehra S, Mohite GM, Ghosh SK, et al. p53 amyloid formation leading to its loss of function: implications in cancer pathogenesis. Cell Death Differ. 2017; 24:1784–98. 10.1038/cdd.2017.105 28644435 PMC5596421

[84] Nicolai S, Rossi A, Di Daniele N, Melino G, Annicchiarico-Petruzzelli M, Raschellà G. DNA repair and aging: the impact of the p53 family. Aging (Albany NY). 2015; 7:1050–65. 10.18632/aging.100858 26668111 PMC4712331

[85] Seitz SJ, Schleithoff ES, Koch A, Schuster A, Teufel A, Staib F, Stremmel W, Melino G, Krammer PH, Schilling T, Müller M. Chemotherapy-induced apoptosis in hepatocellular carcinoma involves the p53 family and is mediated via the extrinsic and the intrinsic pathway. Int J Cancer. 2010; 126:2049–66. 19711344 10.1002/ijc.24861

[86] Charni M, Aloni-Grinstein R, Molchadsky A, Rotter V. p53 on the crossroad between regeneration and cancer. Cell Death Differ. 2017; 24:8–14. 10.1038/cdd.2016.117 27768121 PMC5260496

[87] Amelio I, Knight RA, Lisitsa A, Melino G, Antonov AV. p53MutaGene: an online tool to estimate the effect of p53 mutational status on gene regulation in cancer. Cell Death Dis. 2016; 7:e2148. 10.1038/cddis.2016.42 26986515 PMC4823943

[88] Levine AJ, Tomasini R, McKeon FD, Mak TW, Melino G. The p53 family: guardians of maternal reproduction. Nat Rev Mol Cell Biol. 2011; 12:259–65. 10.1038/nrm3086 21427767

[89] De Laurenzi V, Melino G. Evolution of functions within the p53/p63/p73 family. Ann N Y Acad Sci. 2000; 926:90–100. 10.1111/j.1749-6632.2000.tb05602.x 11193045

[90] Memmi EM, Sanarico AG, Giacobbe A, Peschiaroli A, Frezza V, Cicalese A, Pisati F, Tosoni D, Zhou H, Tonon G, Antonov A, Melino G, Pelicci PG, Bernassola F. p63 Sustains self-renewal of mammary cancer stem cells through regulation of Sonic Hedgehog signaling. Proc Natl Acad Sci USA. 2015; 112:3499–504. 10.1073/pnas.1500762112 25739959 PMC4372004

[91] Novelli F, Lena AM, Panatta E, Nasser W, Shalom-Feuerstein R, Candi E, Melino G. Allele-specific silencing of EEC p63 mutant R304W restores p63 transcriptional activity. Cell Death Dis. 2016; 7:e2227. 10.1038/cddis.2016.118 27195674 PMC4917656

[92] Rivetti di Val Cervo P, Lena AM, Nicoloso M, Rossi S, Mancini M, Zhou H, Saintigny G, Dellambra E, Odorisio T, Mahé C, Calin GA, Candi E, Melino G. p63-microRNA feedback in keratinocyte senescence. Proc Natl Acad Sci USA. 2012; 109:1133–38. 10.1073/pnas.1112257109 22228303 PMC3268329

[93] Melino G, De Laurenzi V, Vousden KH. p73: Friend or foe in tumorigenesis. Nat Rev Cancer. 2002; 2:605–15. 10.1038/nrc861 12154353

[94] Candi E, Schmidt R, Melino G. The cornified envelope: a model of cell death in the skin. Nat Rev Mol Cell Biol. 2005; 6:328–40. 10.1038/nrm1619 15803139

[95] Viticchiè G, Agostini M, Lena AM, Mancini M, Zhou H, Zolla L, Dinsdale D, Saintigny G, Melino G, Candi E. p63 supports aerobic respiration through hexokinase II. Proc Natl Acad Sci USA. 2015; 112:11577–82. 10.1073/pnas.1508871112 26324887 PMC4577137

[96] Candi E, Smirnov A, Panatta E, Lena AM, Novelli F, Mancini M, Viticchiè G, Piro MC, Di Daniele N, Annicchiarico-Petruzzelli M, Melino G. Metabolic pathways regulated by p63. Biochem Biophys Res Commun. 2017; 482:440–44. 10.1016/j.bbrc.2016.10.094 28212728

[97] Candi E, Terrinoni A, Rufini A, Chikh A, Lena AM, Suzuki Y, Sayan BS, Knight RA, Melino G. p63 is upstream of IKK alpha in epidermal development. J Cell Sci. 2006; 119:4617–22. . http://jcs.biologists.org/cgi/doi/10.1242/jcs.03265 10.1242/jcs.03265 17093266

[98] Martin SE, Temm CJ, Goheen MP, Ulbright TM, Hattab EM. Cytoplasmic p63 immunohistochemistry is a useful marker for muscle differentiation: an immunohistochemical and immunoelectron microscopic study. Mod Pathol. 2011; 24:1320–26. 10.1038/modpathol.2011.89 21623385

[99] Compagnone M, Gatti V, Presutti D, Ruberti G, Fierro C, Markert EK, Vousden KH, Zhou H, Mauriello A, Anemone L, Bongiorno-Borbone L, Melino G, Peschiaroli A. ΔNp63-mediated regulation of hyaluronic acid metabolism and signaling supports HNSCC tumorigenesis. Proc Natl Acad Sci USA. 2017; 114:13254–59. 10.1073/pnas.1711777114 29162693 PMC5740608

[100] Di Franco S, Sala G, Todaro M. p63 role in breast cancer. Aging (Albany NY). 2016; 8:2256–57. 10.18632/aging.101042 27783565 PMC5115884

[101] Flores ER, Lozano G. The p53 family grows old. Genes Dev. 2012; 26:1997–2000. 10.1101/gad.202648.112 22987633 PMC3444725

[102] Flores ER, Sengupta S, Miller JB, Newman JJ, Bronson R, Crowley D, Yang A, McKeon F, Jacks T. Tumor predisposition in mice mutant for p63 and p73: evidence for broader tumor suppressor functions for the p53 family. Cancer Cell. 2005; 7:363–73. 10.1016/j.ccr.2005.02.019 15837625

[103] Latina A, Viticchiè G, Lena AM, Piro MC, Annicchiarico-Petruzzelli M, Melino G, Candi E. ΔNp63 targets cytoglobin to inhibit oxidative stress-induced apoptosis in keratinocytes and lung cancer. Oncogene. 2016; 35:1493–503. 10.1038/onc.2015.222 26096935

[104] Niklison-Chirou MV, Steinert JR, Agostini M, Knight RA, Dinsdale D, Cattaneo A, Mak TW, Melino G. TAp73 knockout mice show morphological and functional nervous system defects associated with loss of p75 neurotrophin receptor. Proc Natl Acad Sci USA. 2013; 110:18952–57. 10.1073/pnas.1221172110 24190996 PMC3839698

[105] Rufini A, Niklison-Chirou MV, Inoue S, Tomasini R, Harris IS, Marino A, Federici M, Dinsdale D, Knight RA, Melino G, Mak TW. TAp73 depletion accelerates aging through metabolic dysregulation. Genes Dev. 2012; 26:2009–14. 10.1101/gad.197640.112 22987635 PMC3444727

[106] Tomasini R, Tsuchihara K, Wilhelm M, Fujitani M, Rufini A, Cheung CC, Khan F, Itie-Youten A, Wakeham A, Tsao MS, Iovanna JL, Squire J, Jurisica I, et al. TAp73 knockout shows genomic instability with infertility and tumor suppressor functions. Genes Dev. 2008; 22:2677–91. 10.1101/gad.1695308 18805989 PMC2559903

[107] Amelio I, Inoue S, Markert EK, Levine AJ, Knight RA, Mak TW, Melino G. TAp73 opposes tumor angiogenesis by promoting hypoxia-inducible factor 1α degradation. Proc Natl Acad Sci USA. 2015; 112:226–31. 10.1073/pnas.1410609111 25535359 PMC4291637

[108] Agostini M, Romeo F, Inoue S, Niklison-Chirou MV, Elia AJ, Dinsdale D, Morone N, Knight RA, Mak TW, Melino G. Metabolic reprogramming during neuronal differentiation. Cell Death Differ. 2016; 23:1502–14. 10.1038/cdd.2016.36 27058317 PMC5072427

[109] Tóth B, Garabuczi E, Sarang Z, Vereb G, Vámosi G, Aeschlimann D, Blaskó B, Bécsi B, Erdõdi F, Lacy-Hulbert A, Zhang A, Falasca L, Birge RB, et al. Transglutaminase 2 is needed for the formation of an efficient phagocyte portal in macrophages engulfing apoptotic cells. J Immunol. 2009; 182:2084–92. 10.4049/jimmunol.0803444 19201861

[110] Agostini M, Tucci P, Steinert JR, Shalom-Feuerstein R, Rouleau M, Aberdam D, Forsythe ID, Young KW, Ventura A, Concepcion CP, Han YC, Candi E, Knight RA, et al. microRNA-34a regulates neurite outgrowth, spinal morphology, and function. Proc Natl Acad Sci USA. 2011; 108:21099–104. 10.1073/pnas.1112063108 22160706 PMC3248521

[111] Agostini M, Tucci P, Killick R, Candi E, Sayan BS, Rivetti di Val Cervo P, Nicotera P, McKeon F, Knight RA, Mak TW, Melino G. Neuronal differentiation by TAp73 is mediated by microRNA-34a regulation of synaptic protein targets. Proc Natl Acad Sci USA. 2011; 108:21093–98. 10.1073/pnas.1112061109 22160687 PMC3248477

[112] Martin-Lopez M, Maeso-Alonso L, Fuertes-Alvarez S, Balboa D, Rodríguez-Cortez V, Weltner J, Diez-Prieto I, Davis A, Wu Y, Otonkoski T, Flores ER, Menéndez P, Marques MM, Marin MC. p73 is required for appropriate BMP-induced mesenchymal-to-epithelial transition during somatic cell reprogramming. Cell Death Dis. 2017; 8:e3034. 10.1038/cddis.2017.432 28880267 PMC5636977

[113] Matin RN, Chikh A, Chong SL, Mesher D, Graf M, Sanza’ P, Senatore V, Scatolini M, Moretti F, Leigh IM, Proby CM, Costanzo A, Chiorino G, et al. p63 is an alternative p53 repressor in melanoma that confers chemoresistance and a poor prognosis. J Exp Med. 2013; 210:581–603. 10.1084/jem.20121439 23420876 PMC3600906

[114] Schmitz AA, Govek EE, Böttner B, Van Aelst L. Rho GTPases: signaling, migration, and invasion. Exp Cell Res. 2000; 261:1–12. 10.1006/excr.2000.5049 11082269

[115] Nagano M, Hoshino D, Koshikawa N, Akizawa T, Seiki M. Turnover of focal adhesions and cancer cell migration. Int J Cell Biol. 2012; 2012:310616. 10.1155/2012/310616 22319531 PMC3272802

[116] Halaoui R, McCaffrey L. Rewiring cell polarity signaling in cancer. Oncogene. 2015; 34:939–50. 10.1038/onc.2014.59 24632617

[117] Sgrò F, Bianchi FT, Falcone M, Pallavicini G, Gai M, Chiotto AM, Berto GE, Turco E, Chang YJ, Huttner WB, Di Cunto F. Tissue-specific control of midbody microtubule stability by Citron kinase through modulation of TUBB3 phosphorylation. Cell Death Differ. 2016; 23:801–13. 10.1038/cdd.2015.142 26586574 PMC4832100

[118] Wu DW, Lee MC, Wang J, Chen CY, Cheng YW, Lee H. DDX3 loss by p53 inactivation promotes tumor malignancy via the MDM2/Slug/E-cadherin pathway and poor patient outcome in non-small-cell lung cancer. Oncogene. 2014; 33:1515–26. 10.1038/onc.2013.107 23584477

[119] Schubert J, Brabletz T. p53 Spreads out further: suppression of EMT and stemness by activating miR-200c expression. Cell Res. 2011; 21:705–07. 10.1038/cr.2011.62 21483453 PMC3203673

[120] Kim T, Veronese A, Pichiorri F, Lee TJ, Jeon YJ, Volinia S, Pineau P, Marchio A, Palatini J, Suh SS, Alder H, Liu CG, Dejean A, Croce CM. p53 regulates epithelial-mesenchymal transition through microRNAs targeting ZEB1 and ZEB2. J Exp Med. 2011; 208:875–83. 10.1084/jem.20110235 21518799 PMC3092351

[121] Cianfrocca R, Rosanò L, Tocci P, Sestito R, Caprara V, Di Castro V, De Maria R, Bagnato A. Blocking endothelin-1-receptor/β-catenin circuit sensitizes to chemotherapy in colorectal cancer. Cell Death Differ. 2017; 24:1811–20. 10.1038/cdd.2017.121 28708138 PMC5596423

[122] Delaidelli A, Negri GL, Jan A, Jansonius B, El-Naggar A, Lim JK, Khan D, Zarni Oo H, Carnie CJ, Remke M, Maris JM, Leprivier G, Sorensen PH. MYCN amplified neuroblastoma requires the mRNA translation regulator eEF2 kinase to adapt to nutrient deprivation. Cell Death Differ. 2017; 24:1564–76. 10.1038/cdd.2017.79 28574509 PMC5563988

[123] Bao W, Strömblad S. Integrin alphav-mediated inactivation of p53 controls a MEK1-dependent melanoma cell survival pathway in three-dimensional collagen. J Cell Biol. 2004; 167:745–56. 10.1083/jcb.200404018 15557124 PMC2172581

[124] Mak AS. p53 in cell invasion, podosomes, and invadopodia. Cell Adhes Migr. 2014; 8:205–14. 10.4161/cam.27841 24714032 PMC4198344

[125] Di J, Huang H, Wang Y, Qu D, Tang J, Cheng Q, Lu Z, Zhang Y, Zheng J. p53 target gene Rap2B regulates the cytoskeleton and inhibits cell spreading. J Cancer Res Clin Oncol. 2015; 141:1791–98. 10.1007/s00432-015-1948-8 25762091 PMC11823724

[126] Heiss NS, Gloeckner G, Bächner D, Kioschis P, Klauck SM, Hinzmann B, Rosenthal A, Herman GE, Poustka A. Genomic structure of a novel LIM domain gene (ZNF185) in Xq28 and comparisons with the orthologous murine transcript. Genomics. 1997; 43:329–38. 10.1006/geno.1997.4810 9268636

[127] Dawid IB, Breen JJ, Toyama R. LIM domains: multiple roles as adapters and functional modifiers in protein interactions. Trends Genet. 1998; 14:156–62. 10.1016/S0168-9525(98)01424-3 9594664

[128] Bach I. The LIM domain: regulation by association. Mech Dev. 2000; 91:5–17. 10.1016/S0925-4773(99)00314-7 10704826

[129] Furukawa D, Chijiwa T, Matsuyama M, Mukai M, Matsuo EI, Nishimura O, Kawai K, Suemizu H, Nakagohri T, Ozawa S, Shimada K, Hiraoka N, Nakamura M. Plasma membrane expression of ZNF185 is a prognostic factor in pancreatic ductal carcinoma. Oncol Lett. 2017; 14:3633–40. 10.3892/ol.2017.6633 28927124 PMC5587964

[130] Furukawa D, Chijiwa T, Matsuyama M, Mukai M, Matsuo EI, Nishimura O, Kawai K, Suemizu H, Hiraoka N, Nakagohri T, Yasuda S, Nakamura M. Zinc finger protein 185 is a liver metastasis-associated factor in colon cancer patients. Mol Clin Oncol. 2014; 2:709–13. 10.3892/mco.2014.298 25054034 PMC4106664

[131] Vanaja DK, Cheville JC, Iturria SJ, Young CY. Transcriptional silencing of zinc finger protein 185 identified by expression profiling is associated with prostate cancer progression. Cancer Res. 2003; 63:3877–82. 12873976

[132] Gonzalez HE, Gujrati M, Frederick M, Henderson Y, Arumugam J, Spring PW, Mitsudo K, Kim HW, Clayman GL. Identification of 9 genes differentially expressed in head and neck squamous cell carcinoma. Arch Otolaryngol Head Neck Surg. 2003; 129:754–59. 10.1001/archotol.129.7.754 12874078

[133] Medina PP, Carretero J, Ballestar E, Angulo B, Lopez-Rios F, Esteller M, Sanchez-Cespedes M. Transcriptional targets of the chromatin-remodelling factor SMARCA4/BRG1 in lung cancer cells. Hum Mol Genet. 2005; 14:973–82. 10.1093/hmg/ddi091 15731117

[134] Krøigård AB, Larsen MJ, Lænkholm AV, Knoop AS, Jensen JD, Bak M, Mollenhauer J, Thomassen M, Kruse TA. Identification of metastasis driver genes by massive parallel sequencing of successive steps of breast cancer progression. PLoS One. 2018; 13:e0189887. 10.1371/journal.pone.0189887 29293529 PMC5749725

[135] Wang J, Huang HH, Liu FB. ZNF185 inhibits growth and invasion of lung adenocarcinoma cells through inhibition of the akt/gsk3β pathway. J Biol Regul Homeost Agents. 2016; 30:683–91. 27655485

[136] Oti M, Kouwenhoven EN, Zhou H. Genome-wide p63-regulated gene expression in differentiating epidermal keratinocytes. Genom Data. 2015; 5:159–63. 10.1016/j.gdata.2015.06.002 26484246 PMC4584025

[137] Tang Z, Li C, Kang B, Gao G, Li C, Zhang Z. GEPIA: a web server for cancer and normal gene expression profiling and interactive analyses. Nucleic Acids Res. 2017; 45:W98–102. 10.1093/nar/gkx247 28407145 PMC5570223

